# Impacts of Fortifying Nile Tilapia (*Oreochromis niloticus*) Diet with Different Strains of Microalgae on Its Performance, Fillet Quality and Disease Resistance to *Aeromonas hydrophila* Considering the Interplay between Antioxidant and Inflammatory Response

**DOI:** 10.3390/antiox11112181

**Published:** 2022-11-03

**Authors:** Doaa Ibrahim, Marwa I. Abd El-Hamid, Mayasar I. Al-Zaban, Mohamed ElHady, Mona M. El-Azzouny, Tamer Mohamed ElFeky, Gehan M. Al Sadik, Omima M. Samy, Thoria A. Hamed, Fauzeya Mateq Albalwe, Muneefah Abdullah Alenezi, Anaam E. Omar

**Affiliations:** 1 Department of Nutrition and Clinical Nutrition, Faculty of Veterinary Medicine, Zagazig University, Zagazig 44511, Egypt; 2 Department of Microbiology, Faculty of Veterinary Medicine, Zagazig University, Zagazig 44511, Egypt; 3 Department of Biology, College of Science, Princess Nourah Bint Abdulrahman University, P.O. Box 84428, Riyadh 11671, Saudi Arabia; 4 Department of Fish Diseases and Management, Faculty of Veterinary Medicine, Zagazig University, Zagazig 44511, Egypt; 5 Department of Bacteriology, Animal Health Research Institute (AHRI), Zagazig Branch, Agriculture Research Center (ARC), Zagazig 44511, Egypt; 6 Department of Bacteriology, Animal Health Research Institute (AHRI), Mansura Lab, Agriculture Research Center (ARC), Mansura 35516, Egypt; 7 Department of Pathology and Clinical Pathology, Animal Health Research Institute (AHRI), Zagazig Branch, Agriculture Research Center (ARC), Zagazig 44511, Egypt; 8 Department of Biochemistry, Animal Health Research Institute (AHRI), Zagazig Branch, Agriculture Research Center (ARC), Zagazig 44511, Egypt; 9 Department of Biology, Faculty of Science, Tabuk University, Tabuk 71491, Saudi Arabia

**Keywords:** oxidative stress, microalgae, *Nile tilapia*, performance, antioxidant stability, immunomodulation, *Aeromonas hydrophila*

## Abstract

The oxidative stress facing fish during intensive production brings about diseases and mortalities that negatively influence their performance. Along with that, the increased awareness of omega-3 polyunsaturated fatty acids (omega-3-PUFAs) health benefits has been triggered the introduction of alternative additives in aqua feed that cause not only modulation in fish immune response but also fortification of their fillet. In this context, the role of microalgae mix (NSS) containing *Nannochloropsis oculate* and *Schizochytrium* and *Spirulina* species, which were enriched with bioactive molecules, especially EPA and DHA, was assessed on *Nile tilapia*’s performance, fillet antioxidant stability, immune response, and disease resistance. Varying levels of NSS (0.75%, 1.5%, and 3%) were added to *Nile tilapia*’s diet for 12 weeks and then a challenge of fish with virulent *Aeromonas hydrophila* (*A. hydrophila*) was carried out. Results showed that groups fed NSS, especially at higher levels, showed an improved WG and FCR, which corresponded with enhanced digestive enzymes’ activities. Higher T-AOC was detected in muscle tissues of NSS_3.0%_ fed fish with remarkable reduction in ROS, H_2_O_2_, and MDA contents, which came in parallel with upregulation of *GSH*-*Px*, *CAT*, and *SOD* genes. Notably, the contents of EPA and DHA in fillet were significantly increased with increasing the NSS levels. The mean log_10_ counts of pathogenic *Vibrio* and *Staphylococcus* species were reduced, and conversely, the populations of beneficial *Lactobacillus* and *Bacillus* species were increased more eminent after supplementation of NSS_3.0%_ and NSS_1.5%_. Moreover, regulation of the immune response (lysozyme, IgM, ACH50, NO, and MPO), upregulation of *IL*-*10*, *TGF*-*β*, and *IgM*, and downregulation of *IL*-*1β*, *TNF*-*α*, *HSP70*, and *COX*-*2* were observed following dietary higher NSS levels. After challenge, reduction in *A. hydrophila* counts was more prominent, especially in NSS_3.0%_ supplemented group. Taken together, the current study encourages the incorporation of such microalgae mix in *Nile tilapia*’s diet for targeting maximum performance, superior fillet quality, and protection against *A. hydrophila.*

## 1. Introduction

Due to the intensification of production, *Nile tilapia* (*Oreochromis niloticus*) is usually exposed to multiple biological, physical, environmental, and chemical stressors that can impair their health, reduce their overall performance, and increase their susceptibility to diseases. Moreover, exposure to extrinsic stressors such as changes in water temperature, salinity, pH values, and dissolved oxygen level or chemical toxicants (e.g., heavy metals, fungicides, herbicides, insecticide, etc.) can promote extreme production of reactive oxygen species (ROS), which induces oxidative stress [1,2]. At a high concentration of ROS together with their great reactivity, they can attack the cellular components leading to lipid peroxidation, DNA damage, impaired cell function, and ultimately necrosis or apoptosis [3]. Oxidative stress, identified by cell/tissue injury and attendant oxidative macromolecule damage, results from an imbalance between ROS production and their elimination by protective antioxidant defense mechanisms [4,5]. The cellular antioxidant defense mechanism comprises antioxidant enzymes; superoxide dismutase (SOD), catalase (CAT), glutathione peroxidase (GSH-Px), and other nonenzymatic molecules that can neutralize ROS effects and stabilize cellular functions [1]. Thus, a balance between ROS and cellular antioxidant systems is essential for cell function, regulation, and adaptation to diverse conditions (Nordberg and Arnér, 2001). On the other hand, excessive ROS production can play a vital role in the progression of many inflammatory disorders and regulate various types of transcription factors related to the activation of pro-inflammatory genes [6,7]. To alleviate the impact of stressors and/or to keep the balance between the released ROS/free radicals and cellular antioxidant defense, a great research interest has been focused to find new, safe, and inexpensive dietary supplements with potent antioxidant characteristics [8,9].

Microalgae could be promising feed additives for aquaculture because of their bioactive phytochemicals that exhibit strong antioxidant, anti-inflammatory, and immunomodulatory properties [10]. Recently, increasing attention has focused on microalgae for aquafeeds because of their nutritional quality, especially their elevated fatty acids concentration [11]. Microalgae are relatively high in essential long chain omega-3 polyunsaturated fatty acids (PUFAs) such as docosahexaenoic acid (DHA) and eicosapentaenoic acid (EPA), which are important for maintaining fish health and imparting excellent health benefits to human consumers [11]. Furthermore, utilization of microalgae as hopeful alternatives in combination with diet can aid to improve the immune response and physiological status of larval, juvenile, and adult fish and crustacean species [12]. Additionally, it was proved that microalgae had antimicrobial features against the bacterial fish pathogens, especially *Aeromonas hydrophila* (*A. hydrophila*) [13] owing to the antibacterial components produced by microalgae cells [14]. Virulent *A. hydrophila* is responsible for hemorrhagic septicemia and causes high levels of mortality and significant economic losses in freshwater fish crustaceans and occasionally marine fish [15,16]. This crisis has grown and become more difficult owing to the emergence of multidrug-resistance phenomenon leading to failure in management approaches [17,18,19].

The *Schizochytrium* species, a marine microalga, is recognized as an important, sustainable, and alternative source of oils rich in long-chain omega-3 PUFAs [20]. *Schizochytrium* species contain 18–22% DHA of their dry matter [21] and its supplementation in aquafeed can improve total long-chain omega-3 PUFAs including DHA in the fish fillet [22]. Moreover, *Schizochytrium* species is a prospective source of natural antioxidants as carotenoid and astaxanthin pigments, which could be readily accumulated in fish tissues and strengthen their oxidative stability [11]. Dietary *Schizochytrium* species could maintain/improve the fish lipid metabolism, and their antioxidant, immune, and anti-inflammatory capacities [20].

*Spirulina* is among the widely distributed cultured filamentous microalgae at the commercial scale [23]. Furthermore, feeding on spirulina-enriched diets revealed positive effects on growth performance, carcass composition, immune status, and disease resistance of various fish species [24] owing to its high content of several bioactive molecules with antioxidant and anti-inflammatory activities [24,25].

*Nannochloropsis oculata* (*N. oculata*), an eukaryotic unicellular microalga, is broadly used in aquaculture industry with an important nutritional value due to its elevated contents of proteins and PUFAs, particularly EPA [26]. The feeding of fish on *N. oculata* supplemented diets has possibly been considered to improve the growth performance, feed utilization, immune response, anti-inflammatory activity, antioxidant capacity, and resistance against pathogenic bacterial species [27,28,29] and mitigate the oxidative stress [30].

Although many previous studies have investigated the impacts of dietary application of the three-abovementioned microalgae (*Schizochytrium* and *Spirulina* species and *N. oculata)* separately on fish performance, immune response, oxidative stress, flesh quality, and disease resistance, the current work is, the first, conducted to evaluate the effectiveness of a combination of these microalgae as a dietary supplement for fish. Therefore, we assessed the positive roles of microalgae mix (NSS) on the growth performance and fillet fatty acid profile considering the crosstalk between the oxidative and inflammatory status of *Nile tilapia*. Moreover, their effects on the population of some beneficial and harmful bacteria in addition to their protective roles against *A. hydrophila* challenge in *Nile tilapia* were explored. 

## 2. Materials and Methods

### 2.1. Ethical Approval

All experimental techniques were accompanied in agreement with the rules and recommendations permitted by the Institutional Animal Care and Use Committee (IACUC), Faculty of Veterinary Medicine, Zagazig University, Egypt, under the reference number of ZUIACUC/2F/197/2022.

### 2.2. Fish Maintenance

Four hundred uniformly sized *Nile tilapia*; *Oreochromis niloticus* (*O. niloticus*) weighing 23.87 ± 0.5 g were procured from El-Abassa Fish Hatchery, Sharkia, Egypt. They were then transported to the Fish Research Unit at Faculty of Veterinary Medicine, Zagazig University, Egypt. Prior to the beginning of the experiment, fish were acclimated to the laboratory rearing conditions for two weeks and received the control diet twice daily (Table 1). After that, the experimental fish were allocated in 20 glass aquaria; 20 fish per aquarium and each aquarium was supplemented with dechlorinated tap water. Along the acclimation and experiment periods, all aquaria were kept in constant rearing conditions involving dissolved oxygen (6.7 ± 0.5 mg/L), which was adjusted via an oxygen meter (YSI Company model 56, Yellow Springs, OH, USA), pH (7.2 ± 0.1), which was measured by pH meter (Orion, Thermo Fisher, San Francisco, CA, USA), temperature (24 ± 2 °C), nitrate (5.4 mg/L), nitrite (0.034 mg/L), ammonium (0.23 mg/L), and photoperiod (12 h light: 12 h darkness). Moreover, the water quality parameters recommended by the American Public Health Association were considered.

### 2.3. Microalgae and Diets Formulation

*Nannochloropsis oculate* and *Schizochytrium*, and *Spirulina* species dried powders were provided by National Research Centre, Cairo, Egypt. Four experimental diets were prepared at the Fish Research Unit, Faculty of Veterinary Medicine, Zagazig University, Egypt. The three microalgae (NSS) were added together as a microalgae mixture containing equal proportions (1:1:1) of three different levels (0.25%, 0.5%, and 1%) to make a final concentration of 0.75%, 1.5%, and 3% (*w/w*) in the experimental diets. A microalgae-free diet was prepared and used as a control. The formulation and chemical composition of the diets are shown in Table 1. Feed ingredients were ground with thorough mixing and then water was added to make homogeneous dough. The diets were pelleted (2 mm diameter) using an electric meat mincer, air-dried at room temperature, and then kept in sealed dry plastic bags at 4 °C until use.

### 2.4. Experimental Design

After the two-week acclimation period, fish were randomly distributed in four groups with five replicates (100 fish per group, 20 fish per replicate). Group 1, namely NSS-0 was fed the microalgae-free diet and kept as a control, whereas groups 2–4, namely NSS-0.75, NSS-1.5, and NSS-3 were fed the diets supplemented with the microalgae mixture at 0.75%, 1.5%, and 3%, respectively. Fish were fed until apparent satiation twice daily (9:00 AM and 3:00 PM) for a period of 12 weeks. Fish were weighed at the beginning of the experiment, and then every two weeks until the end of the experiment (12 weeks) to calculate their mean body weight and the biomass present in each aquarium. Fish excreta were carefully siphoned out daily and nearly 75% water was exchanged every day throughout the experiment period.

### 2.5. Growth Performance

Growth and feed performance parameters were assessed basing on initial weight (W_i_), final weight (W_f_), weight gain (WG), specific growth rate (SGR), and feed conversion ratio (FCR), as described formerly [8,31,32]. The following formulas were used:

WG (g) = W_f_, g − W_i_, g

SGR (%/day) = [(Ln W_f_ − Ln W_i_)/t] × 100,

where (Ln W_f_) and (Ln W_i_) are the natural logarithm of final and initial weights (g), respectively, and (t) is the experiment period (days)

FCR = Feed intake, g/WG, g

Protein efficiency ratio (PER) = WG, g/protein intake, g

Survival rate, % = (fish number at the end of experiment /initial fish number) × 100

### 2.6. Blood and Tissue Sampling

At the end of the experiment, five fish from each replicate of the different experimental groups (25 fish per group) were randomly selected for sampling. Blood samples were collected in two different tubes. The first one contained an anticoagulant and the blood in this tube was used to determine the white blood cells (WBCs), red blood cells (RBCs), hematocrit (Ht), and hemoglobin (Hb) concentrations according to Blaxhall and Daisley [33]. The second tube, which did not contain any anticoagulant, was left at room temperature for 2 h allowing the blood to clot, and then it was centrifuged at 1400× *g* for 10 min to obtain serum, which was kept at −20 °C until use for subsequent biochemical and immunological analysis. Furthermore, fresh samples of fish spleen, and musculature were immediately taken from euthanized fish following the guidelines for the Use of Fishes in Research [34] and then stored at −80 °C until use.

### 2.7. Digestive and Liver Enzymes’ Activities

Using commercially available kits acquired from Sigma-Aldrich (Sigma-Aldrich, St. Louis, MO, USA), amylase, chymotrypsin, protease, and lipase were analyzed following the manufacturers’ instructions. Activities of aspartate aminotransferase (AST) and alanine aminotransferase (ALT) were determined as affirmed by the protocols of Reitman and Frankel [35]. 

### 2.8. Fatty Acid Profile and Oxidative/Antioxidant Status in Serum and Muscle Tissues

Extraction of lipid from fish musculature samples was done following an earlier method detailed previously [36]. Briefly, 0.5 g of muscle samples were added to 2.5 mL of chloroform, 0.4 mL of water, and 5 mL of methanol, and then the mixture was subjected to mechanical shaking for 1 h. Subsequently, Na_2_SO_4_ solution (1.5%) and chloroform (2.5 mL each) were added and then the prepared mixture was centrifuged for 3 min at 2000× *g*. To prepare fatty acid methyl esters, hexane and methanolic solution were added, and finally, fatty acid analysis was conducted via gas chromatography (Varian, Palo Alto, CA, USA).

Antioxidant enzymes involving superoxide dismutase (SOD), catalase (CAT), and glutathione peroxidase (GSH-Px) were assayed in fish serum following the methods described previously [36]. Serum levels of malondialdehyde (MDA) were estimated using commercial kits (Nanjing Bioengineering Institute, Nanjing, China). Total antioxidant capacity (T-AOC) was determined in fish muscle tissues via equivalent diagnostic kits (Nanjing Jiancheng Bioengineering Institute, China) following the company’s guidelines. To estimate meat ROS contents, an oxidation technique was utilized [37]. Muscle hydrogen peroxide (H_2_O_2_) amounts were calculated adopting the methods described elsewhere [38] and their values were estimated as μmoL/g of tissue. Moreover, malondialdehyde (MDA) values were evaluated in fish muscle through the thiobarbituric acid reaction according to Livingstone et al. [39].

### 2.9. Assessment of Serum Immune-Mediated Biomarkers 

The serum lysozyme activity was measured by a turbidimetric assay depending on the lysis of Gram-positive bacterium *Micrococcus lysodeikticus* [40]. Nitric oxide (NO) level was assayed using the colorimetric method described elsewhere [41,42]. The total myeloperoxidase (MPO) content was estimated adopting the protocol described by Suzuki et al. [43]. Alternative complement pathway activity (ACH_50_) was determined using rabbit red blood cells as target cells for hemolysis following the method defined by Sunyer and Tort [44]. Immunoglobulin M (IgM) was evaluated via an enzyme-linked immunosorbent assay kit (Sigma Aldrich, MO, USA). The serum cortisol amount was determined following the method described previously [45]. C-reactive protein (CRP) was evaluated by latex advanced nephelometry based on phosphocholine interaction [46].

### 2.10. Gene Expression Analysis

The mRNA levels of *SOD*, *CAT*, *GSH*-*Px*, heat shock protein 70, *HSP70* and cyclooxygenase-2, *COX*-*2* genes were assessed in the fish muscle and those of interleukin; *IL*-*1β*, *IL*-*10*, tumor necrosis factor alpha, *TNF*-*α*, *IgM*, and transforming growth factor beta, *TGF*-*β* genes were evaluated in the fish spleen. Total RNA was extracted from the tissue samples using the QIAamp RNeasy Mini Kit (Qiagen, Hilden, Germany) according to the manufacturer’s protocol. RNA concentration was measured using a nanodrop spectrophotometer and gel electrophoresis was used to assess the RNA integrity. Subsequently, RNA was reverse-transcribed into cDNA using QuantiTect Reverse Transcription Kits (Qiagen, Hilden, Germany) following the manufacturer’s instructions. The quantitative reverse-transcription PCR (qRT-PCR) of a housekeeping gene and the target genes was carried out on a Rotor-Gene Q cycler (Qiagen, Hilden, Germany) using specific primers (Table 2). QuantiTect SYBR Green PCR Kits (Qiagen, Hilden, Germany) were used in all reactions. To calculate and analyze the relative gene expression according to the 2^−ΔΔCT^ method [47], the cycle threshold (Ct) values were detected and β-actin was used as the housekeeping gene.

### 2.11. Real-Time PCR for Quantitative Detection of Fish Bacterial Species

Quantification of some beneficial and pathogenic bacterial species including *Lactobacillus*, *Bacillus*, *Vibrio*, and *Staphylococcus* was carried out by quantitative real-time PCR (RT-PCR) technique at 4, 8, and 12 weeks of age. DNA was extracted from intestinal samples of fish (5 per group) using the commercial Qiagen QIAamp DNA kit (Qiagen, Germany) according to the manufacturer’s directions. The concentration and quality of extracted DNA were determined using the Nano Drop TM 2000 spectrophotometer (Thermo Fisher Scientific Inc., Waltham, MA, USA) and purified DNA was stored at −80 °C until further analysis. The populations of the investigated bacterial species were calculated, in triplicate, via RT-PCR assay carried out on Stratagene MX3005P machine using SYBR^®^ Premix Ex Taq™ kit (TaKaRa, Kyoto, Japan) and previously designed *Lactobacillus*, *Vibrio* and *Staphylococcus* species *16S rRNA* and *Bacillus* species *16S*-*23S rRNA* specific primers (Table 2) adopting the manufacturer’s recommendations. To construct standard curves, DNA samples extracted from pure bacterial cultures were ten-fold serially diluted and quantified in real-time PCR runs to detect their related Ct values. The concentrations of target bacterial species in the examined samples were calculated in respect of log_10_ CFU per gram of fish intestine.

### 2.12. Challenge Test

A well-characterized virulent and multidrug-resistant *A. hydrophila* strain isolated from diseased fish was used for the challenge model to evaluate the effectiveness of the microalgae blend. Prior to the challenge, PCR was utilized to verify the identification of *A. hydrophila* strain using one set of primers targeted *gyrB* gene as previously described [53]. The virulence of the challenging strain was confirmed via PCR amplification of aerolysin (*aer*) and haemolysin (*hyl*) virulence genes [54]. Before a challenge test, fish were examined to be free from *A. hydrophila* infection.

To reveal the in vivo effect of the microalgae blend supplementation on *A. hydrophila* infection, 15 fish/replicate were injected with *A. hydrophila* culture at the median lethal dose, via intraperitoneal injection (0.2 mL/fish) after the end of feeding trial (12 weeks) as previously stated [5,55]. Injected fish were kept under observation for two weeks from the day of *A. hydrophila* injection and immediate clinical signs, post-mortem changes, and mortality were recorded. Liver, kidney, gut, spleen, and skin tissue samples of dead fish were subjected to re-isolation and identification of *A. hydrophila* challenging strain to confirm the presence of *A. hydrophila*. Moreover, quantification of *A. hydrophila* DNA copies in splenic tissue samples was conducted adopting the previously reported protocol at 5, 10, and 15 days post-experimental infection [52].

### 2.13. Statistical Analysis

Statistical analysis was carried out with PASW Statistics 18 (SPSS, Inc., Chicago, IL, USA). The data analysis was conducted using general linear model procedure after testing the homogeneity of variance of the achieved results among experimental fish groups via Levene’s test and normality via Shapiro–Wilk’s test. The Tukey’s test was utilized to detect the significance (*p* < 0.05) among the supplemented groups. The yielded graphs were prepared using GraphPad Prism software (San Diego, CA, USA).

## 3. Results

### 3.1. Effect of NSS on Fish Growth Performance

The dietary addition of a microalgae mixture (NSS) containing equal proportions (1:1:1) of *N. oculate* and *Schizochytrium* and *Spirulina* species for *Nile tilapia* at 0.75, 1.5, and 3% improved their growth performance parameters in a dose-dependent manner (Table 3). Notably, NSS fed groups, especially NSS_3.0%_ and NSS_1.5%_ showed significant (*p* < 0.05) improvements in final body weight (FBW), WG, SGR, and FCR when compared to NSS_0.0%_ group, which was fed the microalgae-free diet. Moreover, NSS_3.0%_ group recorded the most significant (*p* < 0.05) improvements in FBW, WG, FCR, and PER (Table 3). 

### 3.2. Effect of NSS on Digestive and Liver Enzymes

As presented in Table 4, significant (*p* < 0.05) elevations in the levels of chymotrypsin, amylase, and protease digestive enzymes were noted in NSS-received fish compared to non-received control fish (NSS_0.0%_). No significant (*p* > 0.05) differences were detected in the levels of these enzymes among NSS_1.5%_ and NSS_3.0%_ groups, except for protease, which was significantly higher in the NSS_3.0%_ group. No significant (*p* > 0.05) changes were noticed in serum ALT and AST levels among the NSS fed groups and the control one (NSS_0.0%_) (Table 4). 

### 3.3. Effect of NSS on Hematological, Immunological, and Antioxidant Status of Fish

As shown in Table 5, the highest RBCs’ counts were recorded in NSS_1.5%_ and NSS_3.0%_ fed groups. In contrast, the results of other hematological parameters revealed no remarkable variations among NSS fed groups and the control one (NSS_0.0%_). The dietary supplementation of NSS at 0.75%, 1.5%, and 3% significantly (*p* < 0.05) enhanced the serum lysozyme activity in a dose-dependent way when compared to NSS_0.0%_ group, which fed the microalgae-free diet (Table 5). Moreover, NO and ACH_50_ levels were increased with increasing the concentration of NSS mixture. However, only NSS_3.0%_ group exhibited the highest significant (*p* < 0.05) elevation in MPO activity and IgM level and, conversely, the lowest significant (*p* < 0.05) CRP level. Feeding on NSS supplemented diets enhanced the antioxidant defense system of fish (Table 5). Activities of CAT, SOD, and GSH-Px enzymes were prominently (*p* < 0.05) boosted with the increase of dietary NSS content, and the highest antioxidant enzymes’ activities were reported in NSS_3.0%_ group. Correspondingly, the serum level of the lipid peroxidation marker (MDA) was dramatically (*p* < 0.05) reduced in NSS-received groups with the rise of NSS inclusion level. No significant (*p* > 0.05) differences were noticed in serum cortisol levels among the NSS fed groups and the control one (Table 5).

### 3.4. Effect of NSS on Oxidative/Antioxidant Status and Fatty Acid Profile in Muscle Tissues

A noticeable reduction in ROS, H_2_O_2_, and the oxidative stress marker (MDA) levels was observed in the muscle tissues of NSS fed fish, especially with higher levels (Table 6). Furthermore, a significantly (*p* < 0.05) higher T-AOC level was reported in NSS_3.0%_ fed group, followed by NSS_1.5%_ and NSS_0.75%_ groups when compared to the control one (NSS_0.0%_). Influence of NSS supplementation on muscle fatty acid profile (Table 6) revealed that the lowest significant (*p* < 0.05) total saturated fatty acids level (Σ SFAs) was detected in NSS_3.0%_ supplemented group. Moreover, the concentration of total monounsaturated fatty acids (Σ MUSFAs) was remarkably (*p* < 0.05) decreased, and the content of total polyunsaturated fatty acids (Σ PUFAs) was significantly (*p* < 0.05) increased post-supplementation with increasing NSS levels. Another remarkable observation that emerged from data analysis was the significant (*p* < 0.05) dose-dependent elevation in the DHA and EPA contents in NSS fed groups. Correspondingly, the concentration of Σn−3 fatty acid was significantly (*p* < 0.05) increased with increasing the NSS supplementation levels. Inversely, the content of Σn−6 fatty acid and the Σn−6/Σn−3 ratio were remarkably (*p* < 0.05) reduced in all experimental fish groups in a dose-dependent way when compared with the control one (Table 6).

### 3.5. Modulation of Genes Expression by NSS

**Figure 1:**
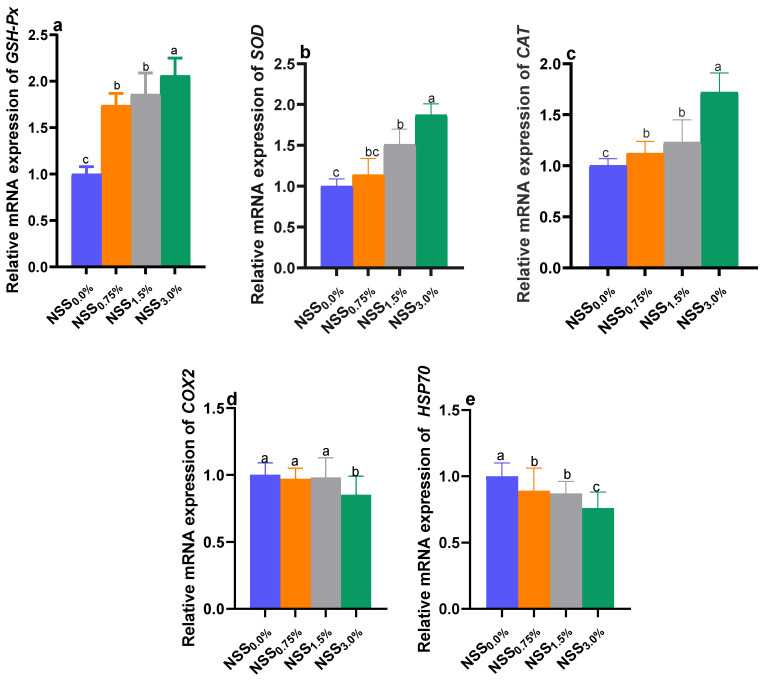
Effect of supplementing diets with varying levels of a microalgae mix (NSS) containing *Nannochloropsis oculate* and *Schizochytrium* and *Spirulina* species at equal proportions (1:1:1) for 12 weeks on relative expression of antioxidant-related genes; *GSH*-*Px*: glutathione peroxidase (**a**), *SOD*: superoxide dismutase (**b**) and *CAT*: catalase (**c**) and stress-related genes; *COX*-*2*: cyclooxygenase-2 (**d**) and *HSP70*: heat shock protein 70 (**e**) in *Nile tilapia* fillet. Data are expressed as means ± SE. Bars with different letters denote significant differences (*p* < 0.05). NSS: microalgae mix containing *Nannochloropsis oculate* and *Schizochytrium* and *Spirulina* species at equal proportions (1:1:1), NSS_0.0%_: control group fed basal diet free from NSS, NSS_0.75%_: basal diet supplemented with 0.75% of NSS, NSS_1.5%_: basal diet supplemented with 1.5% of NSS, NSS_3.0%_: basal diet supplemented with 3% of NSS.

**Figure 2:**
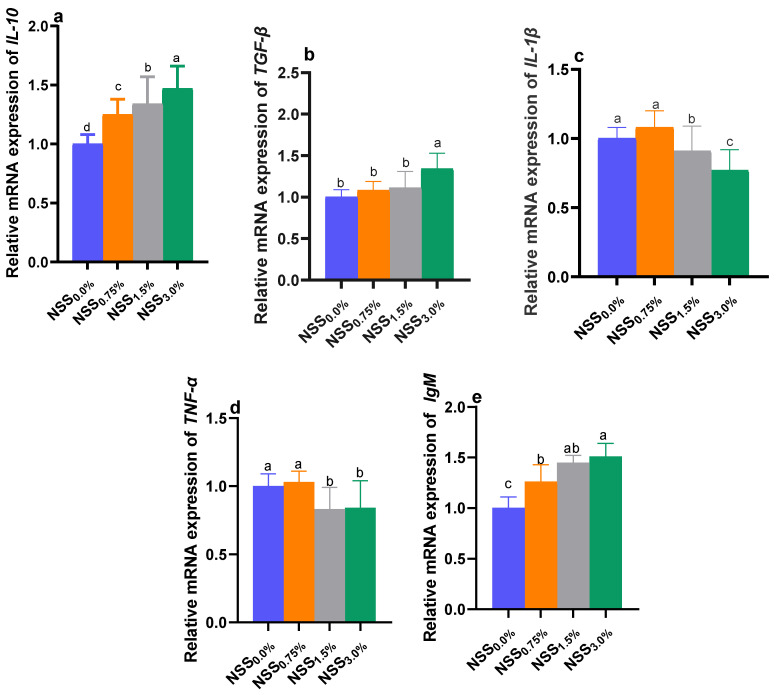
Effect of supplementing diets with varying levels of a microalgae mix (NSS) containing *Nannochloropsis oculate* and *Schizochytrium* and *Spirulina* species at equal proportions (1:1:1) for 12 weeks on relative expression of interleukin *(IL)*-*10* (**a**), transforming growth factor beta; *TGF*-*β* (**b**), *IL*-*1β* (**c**), tumor necrosis factor alpha; *TNF*-*α* (**d**) and Immunoglobulin M; *IgM* (**e**) genes in *Nile tilapia* spleen. Data are expressed as means ± SE. Bars with different letters denote significant differences (*p* < 0.05). NSS: microalgae mix containing *Nannochloropsis oculate* and *Schizochytrium* and *Spirulina* species at equal proportions (1:1:1), NSS_0.0%_: control group fed basal diet free from NSS, NSS_0.75%_: basal diet supplemented with 0.75% of NSS, NSS_1.5%_: basal diet supplemented with 1.5% of NSS, NSS_3.0%_: basal diet supplemented with 3% of NSS.

Dietary NSS administration influenced the relative expression of selected antioxidant, immune-linked, and stress-related genes of *O. niloticus* (Figure 1 and Figure 2). The expression analysis of genes encoding antioxidant enzymes; *CAT*, *SOD*, and *GSH*-*Px* (Figure 1), immunoglobulin M; *IgM* and the anti-inflammatory cytokine; *IL*-*10* (Figure 2) reveled the highest significant (*p* < 0.05) upregulation in NSS_3.0%_ fed fish. Moreover, *TGF*-*β* gene was upregulated significantly (*p* < 0.05) only in the NSS_3.0%_ group unlike the control one. The genes of proinflammatory cytokines; *IL*-*1β* and *TNF*-*α* were significantly (*p* < 0.05) downregulated in NSS_1.5%_ and NSS_3.0%_ fish groups compared to the NSS_0.0%_ one. Meanwhile, the group fed NSS_0.75%_ showed no significant changes in the expression of *IL*-*1β* and *TNF*-*α* genes when compared to the control group (NSS_0.0%_). Notably, the expression of the inflammatory mediator; *COX*-*2* gene, was slightly downregulated in NSS fed groups with no significant variations (*p* > 0.05) compared to the control one (NSS_0.0%_), except for NSS_3.0%_ fish group. The stress-related gene; *HSP70* was significantly (*p* < 0.05) downregulated in fish received NSS supplemented diets when compared to those received the microalgae-free diet. Group NSS_3.0%_ showed a significantly (*p* < 0.05) lower *HSP70* expression rate than other NSS fed groups (NSS_1.5%_ and NSS_0.75%_), which displayed non-significant (*p* > 0.05) variations between each other (Figure 1).

### 3.6. Effect of NNS on Some Intestinal Microbiota

**Figure 3:**
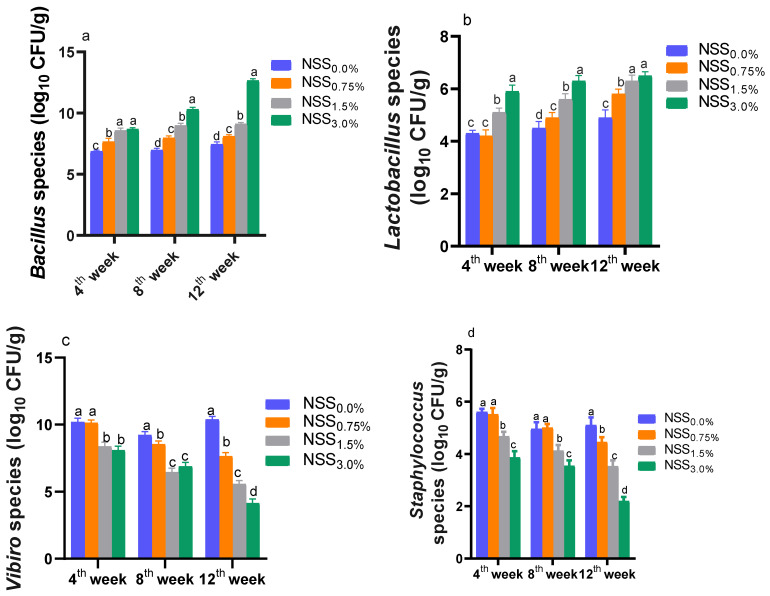
Effect of supplementing diets with varying levels of a microalgae mix (NSS) containing *Nannochloropsis oculate* and *Schizochytrium* and *Spirulina* species at equal proportions (1:1:1) for 12 weeks on the population of some beneficial (*Bacillus*, (**a**) and *Lactobacillus*, (**b**)) and pathogenic (*Vibrio*, (**c**) and *Staphylococcus*, *(***d**)) species in *Nile tilapia* intestinal samples at 4, 8, and 12 weeks of age. Data are expressed as means ± SE. Bars with different letters denote significant differences (*p* < 0.05). NSS: microalgae mix containing *Nannochloropsis oculate* and *Schizochytrium* and *Spirulina* species at equal proportions (1:1:1), NSS_0.0%_: control group fed basal diet free from NSS, NSS_0.75%_: basal diet supplemented with 0.75% of NSS, NSS_1.5%_: basal diet supplemented with 1.5% of NSS, NSS_3.0%_: basal diet supplemented with 3% of NSS.

As illustrated in Figure 3, the inclusion of NSS in fish diet for 12 weeks reduced *Vibrio* and *Staphylococcus* populations and increased *Lactobacillus* and *Bacillus* copies with respect to the control group (NSS_0.0%_). At 4 weeks of age, fish fed NSS_1.5%_ and NSS_3.0%_ had considerable (*p* < 0.05) lower *Vibrio* and *Staphylococcus* counts and higher *Lactobacillus* and *Bacillus* number of copies when compared to the control group with a trend towards significant differences between both levels considering *Lactobacillus* and *Staphylococcus* populations. At 8 weeks of age, dietary supplementation of NSS at different levels increased *Lactobacillus* and *Bacillus* counts in a dose-dependent manner compared to the control group. Meanwhile, statistically significant (*p* < 0.05) decreases in *Vibrio* and *Staphylococcus* numbers in relation to the NSS_0.0%_ group were recorded for NSS_1.5%_ and NSS_3.0%_ and NSS_3.0%_ groups, respectively. At 12 weeks of age, there were dose-dependent rises in *Bacillus* populations and reductions in *Staphylococcus* and *Vibrio* counts post-NSS supplementation in fish diet. Moreover, *Lactobacillus* counts were markedly (*p* < 0.05) increased in NSS_1.5%_ and NSS_3.0%_ groups unlike the control group.

### 3.7. Effect of NNS on Aeromonas Hydrophila Population and Cumulative Mortality Rates

**Figure 4:**
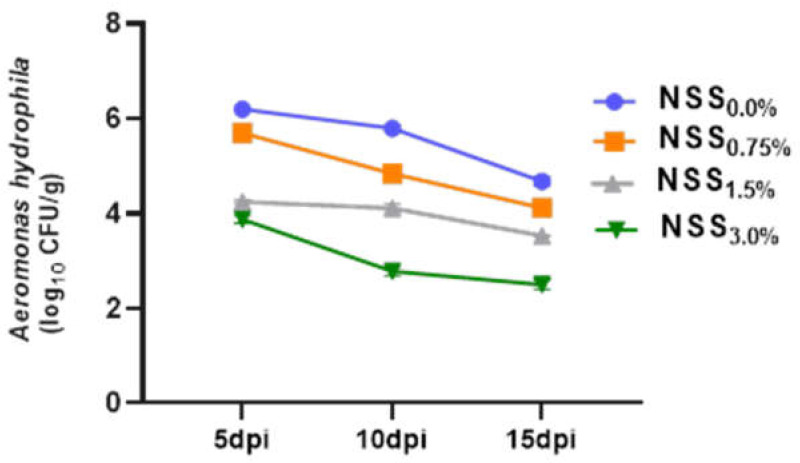
Effect of supplementing diets with varying levels of a microalgae mix (NSS) containing *Nannochloropsis oculate* and *Schizochytrium* and *Spirulina* species at equal proportions (1:1:1) for 12 weeks on the population of *Aeromonas hydrophila* at 5, 10, and 15 days post-infection (dpi). Data are expressed as means ± SE. Bars with different letters denote significant differences (*p* < 0.05). NSS: microalgae mix containing *Nannochloropsis oculate* and *Schizochytrium* and *Spirulina* species at equal proportions (1:1:1), NSS_0.0%_: control group fed basal diet free from NSS, NSS_0.75%_: basal diet supplemented with 0.75% of NSS, NSS_1.5%_: basal diet supplemented with 1.5% of NSS, NSS_3.0%_: basal diet supplemented with 3% of NSS.

**Figure 5:**
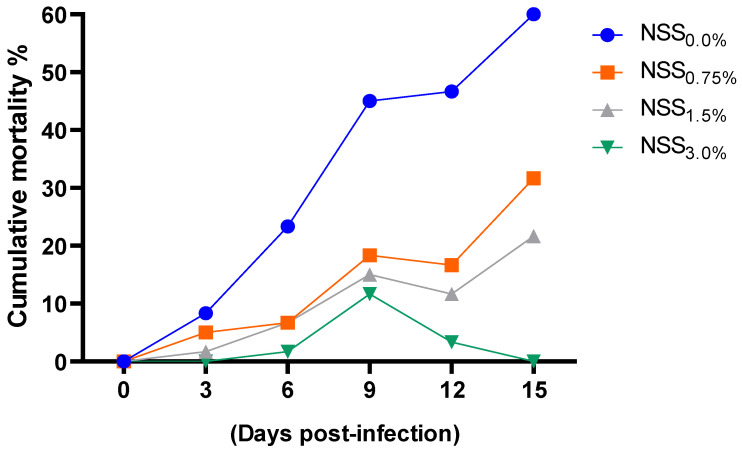
Effect of supplementing diets with varying levels of a microalgae mix (NSS) containing *Nannochloropsis oculate* and *Schizochytrium* and *Spirulina* species at equal proportions (1:1:1) for 12 weeks on the cumulative mortality percentages of *Nile tilapia* after challenge with *Aeromonas hydrophila*. Data are expressed as means ± SE. Bars with different letters denote significant differences (*p* < 0.05). NSS: microalgae mix containing *Nannochloropsis oculate* and *Schizochytrium* and *Spirulina* species at equal proportions (1:1:1), NSS_0.0%_: control group fed basal diet free from NSS, NSS_0.75%_: basal diet supplemented with 0.75% of NSS, NSS_1.5%_: basal diet supplemented with 1.5% of NSS, NSS_3.0%_: basal diet supplemented with 3% of NSS.

As shown in Figure 4 and Figure 5, supplementing fish diet with varying levels of NSS led to a considerable (*p* < 0.05) reduction in *A. hydrophila* counts and cumulative mortality percentages at various time intervals post-infection unlike the free microalgae-challenged group. Of note, fish fed NSS_3.0%_ had the most remarkable (*p* < 0.05) lower *A. hydrophila* counts and cumulative mortality percentages at 15 days post-infection.

## 4. Discussion

Ongoing rapid development of aquaculture has been accompanied by more stressful conditions, which have impaired immune response and augmented disease outbreaks among farmed fish [56]. Concerning these issues that threaten aquaculture industry, the application of natural functional nutrients enriched with omega-3 (n-3) fatty acids not only has significant regulatory effects on inflammatory response in fish [57], but also offers healthy food choice for consumers with numerous health outcomes [58]. It has been shown that many microalgae species have a number of health-promoting impacts, particularly in the prevention and treatment of several diseases owing to their composition from natural antioxidant compounds or production of long-chain PUFAs [59,60,61]. Among these important microalgae are *Nannochloropsis*, *Spirulina*, and *Schizochytrium* species, which are fairly high in n-3 PUFAs such as EPA (C20:5n3) and DHA (C22:6n3), and are important for sustaining fish health and imparting neurological, cardiovascular, and anticancer benefits to humans [11,62,63]. However, the purpose from continuous feeding of such combination of microalgae and investigating the molecular basis clearing their roles on fish health and quality was not properly investigated until now. Therefore, our study is focused on the prospective application of microalgae mix in fish diet to promote its growth and flesh quality and to act as immunostimulant and antioxidant agents with a promising role in protecting against *A. hydrophila* infection. In the current study, after a 12-week feeding period on *N. oculate* and *Schizochytrium* and *Spirulina* species combination enriched with omega-3, we achieved our targeted goal concerning maximum fish production and an enhanced fillet quality with omega-3 (n-3) fatty acids. In accordance, dietary feeding on *Schizochytrium* species for *Nile tilapia* [11] and *Nannochloropsis* species for European sea bass [64] improved their growth rates to certain limits, while feeding of *Nile tilapia* in our study on a combination of selected microalgae enhanced the growth performance parameters of *Nile tilapia* more prominently, especially with increasing their inclusion levels (up to 3%). Besides, more efficient digestion due to higher digestive enzymes’ activities was detected in groups exhibiting improved growth-related parameters and those fed higher levels of microalgae mixtures, which came in the same line with the findings stated previously [65,66,67,68,69]. These positive outcomes in allover growth performance parameters could be attributed to the high contents of microalgae DHA and EPA fatty acids those are linked with the improved health condition of fish, especially those reared under intensive farming conditions [70]. Additionally, DHA and EPA fatty acids are engaged in important roles such as activation of insulin-like growth factor-1 and Akt-mTOR-p70S6K pathway [71] that positively impacted the growth and metabolic regulation of fish. Furthermore, it was proved that improved fish growth due to omega-3 supplementation may be associated with the prompted health condition [70].

Shifting from *Nile tilapia* traditional feeding to those enriched with functional nutrients comes with the same consumers’ needs targeting good quality fillet [72]. Moreover, due to the limited ability of the human body to change alpha-linolenic acid into longer chain omega-3 fatty acids; DHA, EPA, and DPA (less than 10%), it is critical to supply significant amounts of long chain omega-3 fatty acids in the food [73,74]. In the current study, fortification of *Nile tilapia* fillet by n3-PUFAs was prominent in groups fed higher levels of microalgae mixtures enriched with these healthy fatty acids. Our findings of beneficial impacts of including NSS microalgae mixture in *Nile tilapia* diets on deposition of DHA and EPA (two important omega-3 PUFAs) are also consistent with a previous observation [75], where higher PUFAs were observed with increasing the supplementation level of *Schizochytrium* species enriched with DHA fatty acid. Moreover, incorporation of microalgae enriched with DHA and EPA increased their contents in copepods [76]. Additionally, feeding of sea bream on diets enriched with microalgae blends including *Nannochloropsis*, *oculata*, and *Schizocthytrium* species displayed an increased long-chain n-3-PUFAs, DHA, and EPA levels [77]. Obviously, these potential benefits are attributed to the higher contents of n-3 PUFA, which can inhibit LDL-C and VLDL uptake and degradation [78,79]. Similarly, dietary supplementation of omega-3 reduced cholesterol, triglyceride, and VLDL levels [80].

Fish health and immunity are greatly connected to the antioxidant defense system. Exposing fish to stressful conditions those are associated with oxidative stress under intensive farming can trigger the higher ROS production resulting in extensive cell damage. The antioxidant defense system supports fish to retain endogenous ROS at quite minimal levels and to mitigate the oxidative damage provoked by ROS high reactivity [81]. Under normal physiological circumstances, the concentration of free radicals in fish is kept under a dynamic equilibrium due to their constant generation and clearness by its antioxidant system [82]. Conversely, increased ROS production can stimulate cell membranes’ lipids peroxidation and negatively impact fish performance and health [79]. Antioxidant enzymes such as GSH-Px, CAT, and SOD are considered main defense lines against the generation of toxic ROS leading to direct detoxification [83,84]. Fish antioxidant system can be coordinated by dietary enriched antioxidants that can scavenge free radicals. In this context, microalgae are enriched with natural antioxidants; however, searching on the mechanisms by which their impacts on the fish antioxidant system and whether their combination will add an additional benefit for strengthening this function is still scarce and needs more investigation. Herein, activation of antioxidant enzymatic mechanisms in groups fed higher levels of NSS microalgae blend was prominent, as detected by higher serum levels of CAT, SOD, and GSH-Px, and upregulation of their expression in fish muscle. The T-AOC is considered an index to mirror the antioxidant status of the body [85]. Notably, higher T-AOC and reduced fish fillet ROS and H_2_O_2_ levels following supplementation of NSS microalgae mixture implies decreased free radical contents and lipid damage. Similarly, the activities of GSH-Px and SOD antioxidant enzymes’ in the plasma and liver of turbot were enhanced after dietary *Nannochloropsis* species supplementation [86,87]. The higher antioxidant capacity of NSS microalgal mixture in the present study may be attributed to their higher contents of DHA and EPA those possess excellent antioxidant properties [88]. Additionally, increasing dietary levels of omega-3 can reduce ROS production [80] via strengthening cellular ability against oxidative stress. Moreover, *S. platensis* is declared to have pigments those possess antioxidative properties and are capable of scavenging peroxide radicals [89]. On the other hand, higher free radicals result in MDA overproduction, which is one of the end products of lipid peroxidation inside the cells; therefore, the MDA level is generally identified as a marker of oxidative stress [90]. Herein, the contents of MDA in fish fillet were greatly reduced after inclusion of higher levels of NSS microalgae mixture. In accordance, dietary inclusion of algal *Schizochytrium* species augmented the antioxidant status of *Micropterus salmoides* and reduced MDA tissues levels [20]. Taken together, a great deal of researches has claimed the antioxidant functions of several microalgae owing to tocopherols, phenolic compounds, and carotenoids those account for free radical scavenging pursuits supplying a considerable amelioration to oxidative stress responses in different fish species [91,92,93].

An alteration of the redox status and the dysregulation of the immune system during exposure to infectious agents result in an elevation of inflammatory systemic response [94]. Considering that, the inflammatory status prompted by the infectious stimulation is characterized by the reciprocal control of major mediators (COX-2, NO, ROS, and the antioxidant glutathione). COX-2 is an enzyme, which mediates the bioconversion of arachidonic acid to inflammatory prostaglandins with a consequent release of cytokines [40] [95]. After dietary feeding of higher levels of NSS microalgae mixture in our study, an inverse trend was found between the relative expressions of *TGF*-*β* and *IL*-*10* and *TNF*-*α* and *IL*-*B* genes. As evidenced in our study, the regulation of the expression of these inflammatory markers could be mediated by depressing production of ROS and downregulation of *COX*-*2* gene, which are the main messengers those modulate the expression of various genes involved in inflammation [96]. Moreover, lysozymes are ubiquitous defense anti-microbial proteins of the immune system those are associated with the first barrier of innate immunity in fish and have lysis activities against pathogenic bacteria [97]. Additionally, immunoglobulins have very important roles in the defense mechanism via killing microbes and pathogens and restricting the spread of infectious agents [18]. Herein, our consequences cleared that using various levels of NSS microalga mixture enhanced the *Nile tilapia* immune system (IgM, lysozymes, and MPO) prior to the challenge as previously declared elsewhere [30,98]. Similarly, dietary supplementation with microalgae blends comprising *Schizochytrium* species, *Spirulina platensis*, *Chloroella sorokiniana*, and *Chromochloris zofingiensis* significantly decreased the genes expression of pro-inflammatory cytokines; IL-8, IL-6, and IL-1β and increased the lysozyme activity in zebra fish [99]. Moreover, dietary supplementation of 5 or 10% of *S. platensis* significantly boosted lysozyme, serum IgM and total protein levels, thereby enhancing the sturgeon ability to resist various pathogens [100]. Notably, it has been shown that dietary fatty acid composition prompted the non-specific immunity (e.g., serum lysozyme, phagocytosis, and respiratory burst), specific immunity (e.g., antibody production and resistance to pathogens), eicosanoid production, and immune-related genes expression in fish [101,102,103]. Moreover, dietary feeding on omega-3 fatty acids improved immunity of the fish as detected by increasing MPO and total immunoglobulin levels [80], and these positive effects may be related to the reduction in the synthesis of omega-6-derived metabolites, which promote the inflammation [104]. Additionally, omega-3 fatty acids have the ability to reduce inflammation via reducing the production and secretion of cytokines and chemokines by macrophages [105]. Recently, the beneficial roles of dietary omega-3 enriched oils in modulating the expression of cytokines-related genes against mixovirus in marine fish were proved [106]. The feeding of sea bream (*Sparidentex hasta*) on supplemental DHA could enhance the serum immunological parameters like lysozyme and phagocytic activity and modulated the expression of *IL*-*1B*, *IL*-*6*, and *IL*-*10* genes [107]. Furthermore, *Nile tilapia* fed 10% *Nannochloropsis oculata* exhibited significant upregulation of *TGF*-*β* and *IL*-*10* and marked downregulation of *IL*-*1β* and *TNF*-*α* genes [30]. In this regard, the anti-inflammatory properties of *Nannochloropsis oculata* could be attributed to its role as a good potential source of EPA and its high contents of pigments such as zeaxanthin, chlorophyll, astaxanthin, and canthaxanthin [108]. In the same context, *Schizochytrium limacinum* is rich in DHA, which enhances the immune function of white shrimp and golden pompano [109,110]. These positive findings after dietary intake of microalga mixture are resulted from higher contents of n-3-PUFAs, particularly EPA and DHA those could boost an anti-inflammatory environment within the fish body and in that way, they could strengthen its combat against infectious diseases aiming for maximum production [111].

Heat shock proteins (HSPs) are stress-associated keys those play a vital role in adaptive and innate immune responses in fish, and they are strong candidates for the progression of new approaches for preventing the fish diseases [112]. Commonly, HSP70 is expressed in low levels, but its expression rises in reaction to ecological and biological stress conditions [32,113]. Over-expression of *HSP70* gene was observed in sea bream liver tissue post-infection with *Vibrio alginolyticus* [114]. This study denoted that *Nile tilapia* groups fed NSS microalgae blends supplemented diet showed *HSP70* low expression levels. In accordance, the expression levels of *HSP70* were downregulated with increasing levels of *Nannochloropsis oculate* in *Nile tilapia* subjected to air stress [30].

It has been established that gut microbiota heavily affects the health status of aquaculture species regarding digestion, nutrient absorption, immunity, metabolism, and biological antagonism [115]. Microalgae could control the homeostasis of probiotic and harmful bacteria implying a positive impact on the fish health. Regarding beneficial bacteria (*Bacillus* and *Lactobacillus* species), supplementing fish with NSS microalgae blends showed an improvement in their counts with a direct relationship between their populations and high NSS doses, which were illustrated in the form of significant increases as compared to the control group. Numerous studies explored the effects of microalgae such as *Chlorella*, *Tetraselmis*, *Schizochytrium*, and *Nannochloropsis* species on farmed fish microbial ecology [113,116,117]. Kulshreshtha et al. [118] concluded that *Spirulina* species is advantageous for the beneficial intestinal microflora. *Lactobacillus* and *Bacillus* species can be used as growth promoters and immunostimulants as they improved the *Nile tilapia* immune response and disease resistance [119]. Concomitantly, beneficial *Lactobacillus* genus was enriched in zebrafish fed a *Schizochytrium* species supplemented diet [116]. Moreover, other investigators in previous teams [120,121] established positive effects of dietary *Spirulina platensis* to birds on boosting *Lactobacillus* counts in the intestine. On the other hand, dietary supplementation with NSS microalgae blends significantly decreased *Vibrio* counts. Moreover, supplementing diets with the NSS microalgae mixture revealed valuable inhibition against *Staphylococcus* species. The same achievements were reported for *Chlorella salina* and *Tetraselmis chuii* studied previously [13], where they had the most positive records against the fish indicator pathogens such as *Vibrio* species. Concurrently, the results of KoKou et al. [122] demonstrated also that the microalgae *Nannochloropsis* species, *Tetraselmis chuii*, *Isochrysis* species, *Arthrospira platensis*, and *Cocculinella minutissima* cultures inhibited the growth of *Vibrio* species comparing with the control treatments. There were previously similar findings with certain microalgae against *Staphylococcus* species [123].

Infections caused by pathogenic bacteria such as *A. hydrophila* could induce changes in the components of gut microbiota and trigger the malfunction of the physiochemical activities leading to diseases [124]. Moreover, *A. hydrophila* is responsible for hemorrhagic septicemia and causes high levels of mortality and significant economic loss in fish [15,16]. Our results proved the good antibacterial activity of the used NSS microalgae mixture against the challenging *A. hydrophila* strain. Interestingly, *Nile tilapia* supplemented with higher levels of NSS microalgae blend showed lower cumulative mortality rates that came in accordance with the remarkable reduction in *A. hydrophila* counts. These enhanced survival rates could be ascribed to the beneficial effects of NSS microalgae blend on both immune and antioxidant functions of *Nile tilapia*. These finding are in harmony with that of Neveen and Ibraheem [125] suggesting that feeding of microalgae enhances the fish immune response. Thus, the positive effects of adding microalgae in the diet proved to be a practical and simple approach to decrease the pathogenic microbial loads in fish. Antimicrobial features of microalgae cultures have been demonstrated in earlier studies [13,14,122]. These higher antimicrobial activities against the strongest fish pathogens could be attributed to the competition of the bacterial populations associated with microalgae cultures [126] or the production of antibacterial components by microalgal cells. These compounds belonged to various chemical classes such as terpenes, phenols, volatile halogenated hydrocarbons, indoles, fatty acids, and acetogenins [127]. Additionally, the microalgae antimicrobial activity may be related to the antimicrobial proteins, oxygen free radicals and associated microflora produced by microalgae cells [122]. Earlier findings illustrated that DHA and EPA contents and carotenoids could control the immune system of fish in response to invasion of harmful microorganisms [106,128,129]. Our outcomes showed, for the first time, that DHA and EPA components of the used NSS microalgae blend have antibacterial properties against *A. hydrophila*. Likewise, dietary feeding on omega-3 fatty acids decreased the infection against *A. hydrophila* in catfish [80]. Moreover, higher levels of EPA and DHA could inhibit bacterial growth and boost secretion of anti-inflammatory cytokines, thereby protecting zebrafish from *Vibrio vulnificus* infection [130]. It is likely that the high contents of unsaturated fatty acid affect the intestinal membrane structure and function that may influence the attachment sites of the gut mucosa [123].

## 5. Conclusions

Considered together, our results suggested that dietary inclusion of microalgae mix containing *Nannochloropsis oculate* and *Schizochytrium* and *Spirulina* species could display beneficial properties that modulate the composition of the intestinal microbiota and contribute to unique immunomodulation and disease tolerance with consequences for superior fish growth and quality. Therefore, our findings have located the selected microalgae in a unique situation in the aquaculture industry. Despite the outstanding achievement of microalgae mix in protecting the health of fish, there are important challenges to be evaluated considering the proposed mechanisms beyond their beneficial effects. Moreover, it would be very valuable to generalize the positive findings beyond the study’s parameters using microalgae mix. Therefore, conducting more in vivo studies those are required to assess the protective effects of microalgae mix against other pathogenic bacterial species threatening fish farming is an interesting idea for further researches.

## Figures and Tables

**Table 1:** Ingredients and chemical composition of the basal diet.

Item	
Ingredient	%
Fish meal	21.5
Soybean meal	24.00
Yellow corn	33.30
Corn gluten	5.50
Rice bran	10.00
Fish oil	2.80
Lysine	0.10
DL-methionine (98%)	0.20
Threonine	0.10
Di-calcium phosphate	1.20
* Vitamins and minerals premix	1.20
**Chemical analysis**	
Digestible energy (kcal/kg)	2904
Crude protein, %	32.00
Ether extract, %	7.91
Nitrogen free extract, %	45.81
Calcium, %	0.90
Available phosphorus, %	0.45
Lysine, %	2.00
Methionine, %	0.88

* Vitamins and minerals/kg of product: 125 mg biotin, 200 mg folic acid, 28 mg cobalt, 5000 mg pantothenic acid, 2500 mg copper, 0.50 g antioxidant, 75 mg selenium, 17,500 mg zinc, 820 mg iron, 100 mg iodine, 3750 mg manganese, 5000 mg niacin, 1,000,000 IU vitamin A, 1250 mg vitamin B1, 2500 mg vitamin B2, 2485 mg vitamin B6, 3750 mg vitamin B12, 28,000 mg vitamin C, 500,000 IU vitamin D3, 20,000 IU vitamin E, and 500 mg vitamin K.

**Table 2:** Primer sequences utilized for PCR analysis.

Target Gene	Primer Sequence (5′–3′)	Accession Number/Reference
*SOD*	F-GACGTGACAACACAGGTTGC R-TACAGCCACCGTAACAGCAG	XM_003449940.5
*CAT*	F-TCAGCACAGAAGACACAGACA R-GACCATTCCTCCACTCCAGAT	XM_031754288.1
*GSH*-*Px*	F-F-CCAAGAGAACTGCAAGAACGA R-CAGGACACGTCATTCCTACAC	NM_001279711.1
*TGF*-*β*	F-GTTTGAACTTCGGCGGTACTG R-TCCTGCTCATAGTCCCAGAGA	XM_003459454.2
*IL*-*10*	F-CTGCTAGATCAGTCCGTCGAA R-GCAGAACCGTGTCCAGGTAA	XM_013269189.3
*IgM*	F: AGGAGACAGGACTGGAATGCACAA R: GGAGGCAGTATAGGTATCATCCTC	XM_025906584.1
*IL*-*1β*	F-TGCTGAGCACAGAATTCCAG R-GCTGTGGAGAAGAACCAAGC	XM_019365841.2
*TNF*-*α*	F-GAGGTCGGCGTGCCAAGA R-TGGTTTCCGTCCACAGCGT	NM_001279533.1
*HSP70*	F-TGGAGTCCTACGCCTTCAACA R-CAGGTAGCACCAGTGGGCAT	XM_003442456.5
*COX*-*2*	F-GGCCGGGTGTAGTCACAAAT R-CGACCACTACCTACACGCTC	XM_003445052
β-actin	F-CAGCAAGCAGGAGTACGATG R-TGTGTGGTGTGTGGTTGTTTTG	XM_031749543.1
*16s rRNA/*genus *Lactobacillus*	F-TGGAAACAGGTGCTAATACCG R-CCATTGTGGAAGATTCCC	[48]
*16S*-*23S rRNA*/*Bacillus* species	F-GCTGGTTAGAGCGCACGCCTGATA R-CATCCACCGTGCGCCCTTTCTAAC	[49]
*16S rRNA/*genus *Staphylococcus*	F-AACTCTGTTATTAGGGAAGAACA R-CCACCTTCCTCCGGTTTGTCACC	[50]
*16S rRNA*/genus *Vibrio*	F-GGCGTAAAGCGCATGCAGGT R-GAAATTCTACCCCCCTCTACAG	[51]
*ahaI*/*Aeromonas hydrophila*	F-GAGAAGGTGACCACCAAGAACA R-GAGATGTCAGCCTTGTAGAGCT	[52]

*SOD*: superoxide dismutase, *CAT*: catalase, *GSH*-*Px*: glutathione peroxidase, *TGF*-*β*: transforming growth factor beta, *IL*: interleukin, *IgM*: Immunoglobulin M: *TNF*-*α*: tumor necrosis factor alpha, *HSP70*: heat shock protein 70, *COX*-*2*: cyclooxygenase-2.

**Table 3:** Growth performance parameters of *Nile tilapia* (*O. niloticus*) fed diets enriched with different levels of a microalgae mix (NSS) containing *Nannochloropsis oculate* and *Schizochytrium* and *Spirulina* species at equal proportions (1:1:1) for 12 weeks.

Parameter	Experimental Group				*p* Value	SEM
	NSS_0.0%_	NSS_0.75%_	NSS_1.5%_	NSS_3.0%_		
IBW (g/fish	23.90	23.86	23.68	24.05	0.87	0.07
FBW (g/fish)	75.60 ^c^	76.93 ^c^	87.07 ^b^	96.03 ^a^	<0.04	3.02
WG (g/fish)	51.70 ^c^	53.07 ^c^	63.38 ^b^	71.98 ^a^	<0.001	7.50
WG (%)	216.36 ^b^	222.35 ^b^	267.60 ^a^	299.21 ^a^	0.001	16.43
Feed intake (g/fish)	84.96 ^a^	84.57 ^a^	85.13 ^a^	75.70 ^b^	0.03	4.01
FCR	1.65 ^a^	1.60 ^a^	1.34 ^b^	1.05 ^c^	<0.006	0.01
SGR (%)	1.37 ^b^	1.39 ^b^	1.55 ^a^	1.65 ^a^	<0.001	0.00
PER	1.90 ^b^	1.96 ^b^	2.32 ^b^	2.98 ^a^	<0.001	0.02

IBW: initial body weight, FBW: final body weight, WG: weight gain, FCR: feed conversion ratio, SGR: specific growth rate, PER: protein efficiency ratio, SEM: standard error of the mean. Mean values with different letters in the same row differ significantly at *p* < 0.05. NSS: microalgae mix containing *Nannochloropsis oculate* and *Schizochytrium* and *Spirulina* species at equal proportions (1:1:1), NSS_0.0%_: control group fed basal diet free from NSS, NSS_0.75%_: basal diet supplemented with 0.75% of NSS, NSS_1.5%_: basal diet supplemented with 1.5% of NSS, NSS_3.0%_: basal diet supplemented with 3% of NSS.

**Table 4:** Digestive and liver enzymes of *Nile tilapia* (*O. niloticus*) fed diets enriched with different levels of a microalgae mix (NSS) containing *Nannochloropsis oculate* and *Schizochytrium* and *Spirulina* species at equal proportions (1:1:1) for 12 weeks.

Parameter	Experimental Group				*p* Value	SEM
	NSS_0.0%_	NSS_0.75%_	NSS_1.5%_	NSS_3.0%_		
Chymotrypsin (U/L)	24.60 ^c^	26.30 ^b^	27.57 ^a^	27.83 ^a^	0.008	0.03
Amylase (U/L)	28.23 ^c^	30.73 ^b^	32.33 ^a^	32.30 ^a^	0.009	0.26
Lipase (U/L)	25.67 ^b^	26.40 ^ab^	27.83 ^a^	28.43 ^a^	<0.01	0.08
Protease (U/L)	28.47 ^d^	29.27 ^c^	30.53 ^b^	33.20 ^a^	0.02	0.17
ALT (U/L)	60.40	60.03	60.03	59.93	0.68	0.54
AST(U/L)	17.27	17.07	17.40	17.07	0.09	0.16

ALT: alanine transaminase, AST: aspartate transaminase, SEM: standard error of the mean. Mean values with different letters in the same row differ significantly at *p* < 0.05. NSS: microalgae mix containing *Nannochloropsis oculate* and *Schizochytrium* and *Spirulina* species at equal proportions (1:1:1), NSS_0.0%_: control group fed a basal diet free from NSS, NSS_0.75%_: basal diet supplemented with 0.75% of NSS, NSS_1.5%_: basal diet supplemented with 1.5% of NSS, NSS_3.0%_: basal diet supplemented with 3% of NSS.

**Table 5:** Hematological, immunological, and antioxidant markers of *Nile tilapia* (*O. niloticus*) fed diets enriched with different levels of a microalgae mix (NSS) containing *Nannochloropsis oculate* and *Schizochytrium* and *Spirulina* species at equal proportions (1:1:1) for 12 weeks.

Parameter	Experimental Group				*p* Value	SEM
	NSS_0.0%_	NSS_0.75%_	NSS_1.5%_	NSS_3.0%_		
RBCs (×10^6^/μL)	2.36 ^b^	2.38 ^b^	2.41 ^ab^	2.55 ^a^	0.02	0.07
Ht (%)	32.43	33.00	32.55	32.53	0.09	0.57
Hb (g/dL)	7.21	7.37	7.35	7.42	0.11	0.09
WBCs (×10^3^/μL)	6.93	6.81	6.48	6.88	0.08	0.16
Lysozyme (μg/mL)	0.89 ^d^	1.16 ^c^	1.42 ^b^	1.55 ^a^	<0.001	0.13
NO (μmol/L)	0.40 ^c^	0.68 ^b^	0.74 ^b^	0.88 ^a^	<0.001	0.06
ACH_50_ (u/mL)	258.00 ^c^	328.67 ^b^	341.33 ^b^	382.00 ^a^	<0.001	7.16
MPO (μmoL/L)	0.67 ^b^	0.64 ^b^	0.73 ^ab^	0.82 ^a^	<0.001	0.25
IgM (μg/mL)	28.50 ^b^	28.27 ^b^	28.90 ^b^	29.78 ^a^	<0.001	1.38
MDA (nmoL/mL)	9.50 ^a^	8.07 ^b^	6.23 ^c^	4.07 ^d^	<0.001	0.06
CAT (U/L)	78.93 ^c^	90.63 ^b^	93.33 ^ab^	96.53 ^a^	0.02	0.96
SOD (μ/mL)	11.23 ^c^	14.80 ^b^	16.67 ^a^	17.70 ^a^	0.03	0.14
GSH-Px (μmoL/mg)	4.37 ^c^	4.43 ^c^	5.80 ^b^	7.90 ^a^	<0.001	0.04
CRP (ng/mL)	8.97 ^a^	7.1333 ^b^	7.0333 ^b^	5.367 ^c^	<0.001	0.09
Cortisol (nmol/L)	5.88	6.05	5.91	6.00	0.06	0.25

RBCs: red blood cells, Ht: hematocrit, Hb: hemoglobin, WBCs: white blood cells, NO: nitric oxide, ACH_50_: alternative complement pathway activity, MPO: myeloperoxidase, IgM: immunoglobulin M, MDA: malondialdehyde, CAT: catalase, SOD: superoxide dismutase, GSH-Px: glutathione peroxidase, CRP: C-reactive protein, SEM: standard error of the mean. Mean values with different letters in the same row differ significantly at *p* < 0.05. NSS: microalgae mix containing *Nannochloropsis oculate* and *Schizochytrium* and *Spirulina* species at equal proportions (1:1:1), NSS_0.0%_: control group fed basal diet free from NSS, NSS_0.75%_: basal diet supplemented with 0.75% of NSS, NSS_1.5%_: basal diet supplemented with 1.5% of NSS, NSS_3.0%_: basal diet supplemented with 3% of NSS.

**Table 6:** Oxidative/antioxidant status and fatty acid profile in muscle tissues of *Nile tilapia* (*O. niloticus*) fed diets supplemented with varying levels of a microalgae mix (NSS) containing *Nannochloropsis oculate* and *Schizochytrium* and *Spirulina* species at equal proportions (1:1:1) for 12 weeks.

Parameter	Experimental Groups				*p* Value	SEM
	NSS_0.0%_	NSS_0.75%_	NSS_1.5%_	NSS_3.0%_		
MDA (nmol/g tissue)	23.63^a^	22.83 ^ab^	21.80 ^b^	20.73 ^b^	<0.01	0.09
ROS	90.00 ^a^	88.83 ^a^	81.63 ^b^	76.87 ^c^	<0.01	0.90
H_2_O_2_ (μmoL/g tissue)	2.99 ^a^	2.86 ^b^	2.46 ^c^	2.22 ^d^	0.03	1.30
T-AOC (U/mg prot)	1.54 ^c^	1.80 ^b^	1.86 ^b^	2.38 ^a^	0.04	0.17
Σ SFAs	37.60 ^a^	35.11 ^b^	34.12 ^b^	31.92 ^c^	<0.001	0.39
Σ MUSFAs	44.13 ^a^	38.59 ^b^	31.96 ^c^	29.10 ^d^	0.03	0.29
Σ PUFAs	48.69 ^c^	52.05 ^b^	55.62 ^a^	58.90 ^a^	<0.01	0.16
EPA	0.76 ^d^	0.91 ^c^	1.86 ^b^	2.01 ^a^	0.04	1.34
DHA	1.23 ^d^	1.45 ^c^	3.27 ^b^	3.86 ^a^	<0.01	0.96
Σn−3	4.10 ^d^	5.30 ^c^	6.30 ^b^	8.10 ^a^	0.03	0.22
Σn−6	42.69 ^a^	40.25 ^b^	38.69 ^c^	35.4 ^d^	0.01	0.35
Σn−6/Σn−3	7.62 ^a^	5.51 ^b^	4.35 ^c^	2.85 ^d^	0.02	0.17

MDA: malondialdehyde, ROS: reactive oxygen species, H_2_O_2_: hydrogen peroxide, T-AOC: total antioxidant capacity, Σ SFAs: total saturated fatty acids, Σ MUFAs: total monounsaturated fatty acids, Σ PUFAs: total polyunsaturated fatty acids, EPA: eicosapentaenoic acid, DHA: docosahexaenoic acid, SEM: standard error of the mean. Mean values with different letters in the same row differ significantly at *p* < 0.05. NSS: microalgae mix containing *Nannochloropsis oculate* and *Schizochytrium* and *Spirulina* species at equal proportions (1:1:1), NSS_0.0%_: control group fed basal diet free from NSS, NSS_0.75%_: basal diet supplemented with 0.75% of NSS, NSS_1.5%_: basal diet supplemented with 1.5% of NSS, NSS_3.0%_: basal diet supplemented with 3% of NSS.

## Data Availability

The data presented in this study are available upon request from the corresponding author.
